# The Effects of Systemic Tranexamic Acid Administration on Drainage Volume, Duration of Drain Placement, and Length of Hospital Stay in Skin- and Nipple-Sparing Mastectomies with Immediate Expander-Based Breast Reconstruction

**DOI:** 10.3390/jcm13216507

**Published:** 2024-10-30

**Authors:** Leon Guggenheim, Sara Magni, Armin Catic, Alberto Pagnamenta, Yves Harder, Daniel Schmauss

**Affiliations:** 1 Faculty of Biomedical Sciences, Università della Svizzera Italiana, CH-6900 Lugano, Switzerland; leon.guggenheim@eoc.ch (L.G.);; 2 Department of Plastic, Reconstructive and Aesthetic Surgery, Ospedale Regionale di Lugano, Ente Ospedaliero Cantonale (EOC), CH-6900 Lugano, Switzerland; saramagnimd@gmail.com; 3 Clinical Trial Unit (CTU), Ente Ospedaliero Cantonale (EOC), CH-6900 Lugano, Switzerland; alberto.pagnamenta@eoc.ch; 4 Department of Plastic, Reconstructive and Aesthetic Surgery and Hand Surgery, Centre Hospitalier Universitaire Vaudois (CHUV), CH-1011 Lausanne, Switzerland; yves.harder@chuv.ch; 5 Faculty of Biology and Medicine, University of Lausanne (UNIL), CH-1015 Lausanne, Switzerland

**Keywords:** tranexamic acid, systemic tranexamic acid, mastectomy, complications, drainage, expander

## Abstract

**Background**: Skin- (SSM) and nipple-sparing (NSM) mastectomies are frequently performed surgeries with a considerable risk for post-operative hematoma or seroma. Tranexamic acid (TXA) is a potent antifibrinolytic drug commonly used in many surgical fields but rather novel in plastic and, specifically, breast surgery. This study investigates the influence of TXA in patients undergoing SSM or NSM with expander-based reconstruction (EbR) on post-operative outcomes. **Methodology**: A retrospective study was conducted on 132 patients undergoing uni- or bilateral SSM or NSM with EbR between May 2015 and March 2022. Patients receiving systemic TXA treatment for 48 h following a standardized protocol were compared to those who received no treatment. Multivariable linear regression was performed to identify influencing factors and quantify their effect on drainage volume, duration of drain placement, length of hospital stay, post-operative bleeding, and seroma formation. **Results**: The 132 patients underwent a total of 155 mastectomies (72 in the TXA group, 83 in the control group). TXA significantly reduced drainage volume (−22.3 mL, *p* = 0.011). Duration of drain placement and length of hospital stay were significantly shorter in the TXA group (*p* < 0.001 and *p* = 0.001). No significant side effects were reported. **Conclusion**: TXA is a safe drug if administered respecting the well-defined contraindications. Systemic TXA administration significantly reduces drainage volume in patients undergoing SSM or NSM and should encourage surgeons to reconsider using drains in post-operative protocols. Duration of drain placement and length of hospital stay were significantly reduced in the TXA group but other factors like resection weight might have a more substantial impact.

## 1. Introduction

Tranexamic acid (TXA) is one of the most widely used and studied antifibrinolytic drugs in medical practice today. It acts as a lysine analog, blocking lysine receptors on plasminogen, thereby decreasing its conversion to plasmin [1]. Furthermore, it acts directly as an anti-plasmin agent [2]. Plasmin, in turn, is an important enzyme active in the degradation of many blood plasma proteins, including fibrin, which is present in blood clots but also part of inflammatory processes [3,4]. Through this mechanism of action, it can be used to treat blood loss in many situations, first and foremost massive hemorrhage after trauma [5] or post-partum [6]. In recent years, its use in perioperative blood loss prevention has been increasing, and studies have shown decreased mortality and need for blood transfusions post-operatively when TXA was administered [7]. Therefore, it is now considered a standard in many fields, such as orthopedic and cardiothoracic surgery.

Other studies have further investigated its effect on inflammatory pathways and have found that TXA reduces several inflammatory cytokines [8], including IL-10 [9], IL-6 [10,11,12], TNFα [10,11,13], and CRP [12], and reduces the transvasal migration of leukocytes after reperfusion [14]. Furthermore, studies have shown that TXA normalizes vascular permeability after pathological situations, therefore decreasing extravasal fluid accumulation [8,15].

Plastic surgery, including reconstructive and aesthetic procedures, presents a wide range of possible applications of TXA since many operations are associated with extended tissue undermining and respectively a large wound surface. More recently, some evidence has also emerged from both aesthetic and non-aesthetic breast surgery. Literature shows that the application of TXA significantly reduces the incidence of post-operative hematoma in patients undergoing breast surgery, particularly in aesthetic procedures [16]. Furthermore, studies have shown a significant reduction in post-operative drainage volume and length of hospital stay after surgery for breast cancer, including axillary lymph node dissection, lumpectomy, and mastectomy [17,18]. However, the available literature is scarce, especially on the use of TXA in patients undergoing mastectomy.

Mastectomies are associated with a high risk of post-operative complications like hematoma and seroma, with an incidence of 2.3–3.3% [19,20,21] and 6.9–51% [21,22,23], respectively. These complications can lead to surgical revision, increased patient comorbidity, higher infection risk, prolonged hospitalization, and eventually, delay of further oncological treatment [24,25,26,27]. Many mastectomy techniques have been developed over the years, from the radical mastectomy by Halsted [28] through the modified radical [29] and simple mastectomy [30] up to techniques that preserve the skin envelope, including skin-sparing (SSM) [31], nipple-sparing (NSM) [32], and skin-reducing mastectomy (SRM) [33,34], that are common nowadays. These techniques have different indications and present with different complications, and although previous studies have not shown any significant variance in drainage volume between these mastectomy techniques [35,36], the differentiation between skin-sparing (including SSM, NSM, and SRM) and simple/radical mastectomy seems inherent due to the difference in pocket size and thus wound surface.

Although the exact formation process has not yet been fully elucidated, it is known that drainage fluid is composed of both blood and serous fluid, which in turn consists of inflammatory exudates produced as a physiological reaction to surgical trauma through ultrafiltration of the blood. Several studies have identified risk factors for excessive fluid production or seroma formation, which include the type of surgery (breast-conserving vs. mastectomy) [37,38], number and extent of axillar lymph node involvement [39], and extent of dissection [40]. Furthermore, lymphatic leakage following axillar lymph node surgery was identified as a contributing factor to seroma formation [26,41].

In June 2019, we implemented a change in regimen wherein all patients undergoing mastectomy received a 48 h systemic administration of TXA perioperatively, given there are no contraindications. This study aims to evaluate the effect of systemic TXA administration on drainage volume, duration of drain placement, length of hospital stay, and incidence of post-operative complications, namely hematoma and seroma, in patients undergoing mastectomy with immediate expander-based reconstruction (EbR).

## 2. Materials and Methods

### 2.1. Study Design

After approval by the local ethics committee (BASEC number 2021-01918), a retrospective study was performed according to the STROBE guidelines of 132 patients undergoing immediate EbR at a single institution between 2015 and 2022. Inclusion criteria included all female patients over 18 who gave informed consent and who underwent SSM, NSM, or SRM for breast cancer or as a prophylactic intervention in gene carriers or high-risk patients. The exclusion criteria were the following: TXA administration other than the mentioned 48 h protocol, previous surgery on the breast of interest, alterations in preoperative coagulation lab, and pregnancy. TXA was administered in all patients after June 2019 except for patients who had clear contraindications to TXA, i.e., known allergy to TXA, previous thromboembolic events, and active thromboembolic disease.

Data were collected from patient files, including operations reports and drainage volume measurements conducted by ward nurses and checked by resident doctors throughout the hospital stay.

Patients were divided into two groups: The case group (patients operated on after June 2019) included 83 breasts receiving the 48 h TXA protocol (1 g i.v. at the beginning of surgery, followed by 1 g i.v. every eight hours for the first 24 h and 1 g p.o. every eight hours for the following 24 h post-operatively) and 72 breasts in the control group (patients operated on before June 2019) who did not receive TXA.

The primary outcome measure was drainage volume, and the two secondary outcome measures included duration of drain placement and respective length of hospital stay. The mastectomy was performed by one of two senior breast surgeons, while expander implantation was performed in a pre- or sub-pectoral manner by one of two senior plastic surgeons.

For subpectoral placement, an absorbable mesh, cut into two strips, was fixed between the lower border of the pectoralis major muscle and the chest wall to prevent muscle retraction.

Following verification of mastectomy flap perfusion with indocyanine green fluorescence analysis, the flaps were trimmed as necessary, and the expanders were inserted.

Nanotextured expanders were secured at the inferomedial and inferolateral pole using their tabs and a braided, non-absorbable suture, whereas macrotextured expanders required no fixation.

The application of a biological mesh was left to the discretion of the surgeon.

After placement of surgical drains, the wound closure was performed in a layered fashion using absorbable sutures.

A total of 32 variables were extracted (Table 1, Table 2 and Table 3).

Age was calculated as the difference between the date of birth and date of surgery. Body mass index (BMI) was calculated using height and weight before surgery. Comorbidities were extracted from previous medical history and included hypertension, diabetes mellitus (type 1 and 2), heart disease, including arrhythmias, conduction problems, atrial fibrillation, and cardiomyopathies, as well as kidney disease, including chronic renal insufficiency and chronic kidney disease. Current smoking, chronic NSAID use, antiaggregation and anticoagulation therapy, previous thromboembolic events, previous radiotherapy, and neoadjuvant chemotherapy were further extracted from previous medical history. The biochemical variables thrombocytes, QUICK, and international normalized ratio (INR) were also recorded within one year prior to the operation if no confounding factors, such as neoadjuvant chemotherapy, were present. The side of the operated breast (right or left), resection weight, type of mastectomy (skin sparing, nipple sparing, skin reducing), sentinel lymph node biopsy, axillar lymph node dissection, additional axillar access, expander placement (prepectoral/subpectoral), expander type (Allergan Natrelle 133 (AbbVie Inc., North Chicago, IL, USA), Mentor CPX4 (Johnson & Johnson Services, Inc., New Brunswick, NJ, USA), Motiva Flora (Establishment Labs^®^ S.A, Alajuela, Costa Rica)), synthetic mesh application, and biologic mesh (acellular dermal matrix) application were extracted from surgical reports. Axillar lymph node dissection ad continuitatem was defined as patients with axillar lymph node dissection but no additional axillar access.

The complications, hematoma and seroma, were recorded within the first month after surgery. Seroma has been defined as a collection of clear and transparent fluid requiring interventional management, i.e., aspiration under ultrasound guidance or evacuation in the operating room. Drainage volume was measured from the first post-operative day until its removal. To best standardize the time-point of drain removal for the duration of drain placement, the total days until measured fluid production was less than 30 mL/24 h were calculated, and 30 mL was chosen as a common cut-off value for drainage after breast reconstruction with expanders [42]. Length of hospital stay was defined as days from surgery until discharge.

### 2.2. Statistical Analysis

Quantitative data were presented as mean with standard deviation or as median with the corresponding 25th and 75th percentiles. Qualitative data were presented as percentages with absolute numbers. Variables were compared between subjects receiving TXA and subjects not receiving TXA using Student’s *t*-test, Mann–Whitney test, chi-squared test, or Fisher exact test as appropriate.

For our primary outcome, a linear mixed-effects regression model was used due to its ability to account for correlated data (daily measurement of fluid drainage) and the different number of days with drain among subjects. This regression model with random effects on the intercept accounting for subjects permits us to obtain adjusted regression coefficients and identify predictors independently associated with the outcome of interest. It returns the change in drainage per day for each variable.

For our two secondary outcome measures, normal linear regression models were used.

We performed univariable analysis on all variables for our primary and two secondary outcome measures. A fully adjusted model was then built based on the results of univariable regression analysis (cut-off *p*-value < 0.15) and a priori knowledge. The variables included in the full model are age, BMI, current smoking, type of mastectomy, biologic mesh (acellular dermal matrix) application, expander type used, axillar lymph node dissection ad continuitatem, resection weight, hematoma, and TXA.

All statistical tests were performed two-sided, and *p*-value < 0.05 was considered statistically significant. Statistical analysis was performed using Stata version 17.0 software (StataCorp LP, College Station, TX, USA) and R version 4.3.2.

## 3. Results

A total of 141 patients undergoing EbR between 2015 and 2022 were included; 9 were excluded, resulting in a final patient cohort of 132 patients, accounting for 155 breasts. Of those, 83 breasts were in the TXA group, with 72 breasts in the control group. Besides common symptoms like headache and nausea, no significant side effects requiring discontinuation of TXA administration have been described by the patients receiving TXA.

Hematoma and seroma rates did not differ between the two groups, with an incidence of 6% in the TXA group and 5.6% in the control group for hematoma and 6% in the TXA group and 4.2% in the control group for seroma—neither reaching statistical significance (Table 3).

For total drainage volume, after full adjustment, TXA was associated with an average reduction in drainage volume of 19 mL per day (−19, 95%CI −34.96–(−3.02), *p* = 0.020). Resection weight was associated with an increased average drainage volume of 0.04 mL per resected gram per day (0.04, 95%CI 0.01–0.06, *p* = 0.001). Axillar lymph node dissection ad continuitatem with the mastectomy pocket was significantly associated with an average increase in drainage of 18 mL per day (18.3, 95%CI 6.90−29.69, *p* = 0.002). Furthermore, hematoma significantly impacted drainage volume with an average increase of 24 mL per day (23.7, 95%CI −10.58–36.82, *p* < 0.001), while skin-reducing mastectomy was associated with an average reduction of 11 mL per day (−10.8, 95%CI −20.76–(−0.79), *p* = 0.034) compared to skin-sparing mastectomy. The use of Allergan (Natrelle 133) and Motiva (Flora) expanders were associated with a reduced average drainage volume of 18 mL per day (−18.4, 95%CI −34.27–(−2.52), *p* = 0.023) and 24 mL per day (−23.5, 95%CI −33.30–(−13.79), *p* < 0.001), respectively, compared to the Mentor (CPX4) expander (Table 4).

**Figure 1:**
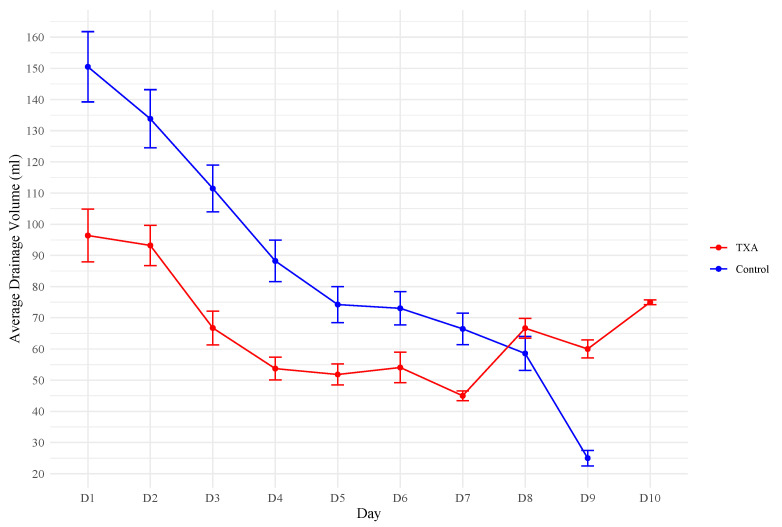
Average drainage volume per day.

The difference in drainage volume between the TXA and control groups was stronger during the first 4 days, specifically throughout the 48 h period of administration (Figure 1).

Drain removal occurred after a mean of 3.3 days (3.33, 95%CI −2.91–3.76) in the TXA group compared to 4.6 days (4.63, 95%CI 4.24–5.01) in the control group (*p* < 0.001) (Table 3). Factors associated with an increase in the duration of drain placement were resection weight with an increase of 0.003 days per gram (0.003, 95%CI 0.001–0.005, *p* = 0.001) and hematoma with an increase of 1.2 days (1.17, 95%CI 0.005–2.33, *p* = 0.049). Motiva (Flora) expanders were associated with a decrease in duration of drain placement of 1.3 days (−1.31, 95%CI −2.20–(−0.42), *p* = 0.004) compared to the Mentor (CPX4) expander (Table 5).

Mean length of hospital stay was 5.3 days (5.28, 95%CI 4.88–5.67) in the TXA group compared to 6.2 days (6.17, 95%CI 5.82–6.51) in the control group (*p* = 0.001) (Table 3). Administration of TXA was not associated with a significant influence on the length of hospital stay (−0.61, 95%CI −1.90–0.67, *p* = 0.349). The two factors significantly associated with an increase in length of hospital stay were BMI with an increase of 0.1 days per kg/m^2^ (0.10, 95%CI 0.003–0.19, *p* = 0.043) and resection weight with an increase of 0.002 days per gram (0.002, 95%CI 0.000–0.004, *p* = 0.025). The Motiva (Flora) expander was associated with a reduction in length of hospital stay of 1.2 days (−1.24, 95%CI −2.01–(−0.46), *p* = 0.002) compared to the Mentor (CPX4) expander (Table 6).

## 4. Discussion

TXA is a potent antifibrinolytic drug commonly used in surgical fields like traumatology, gynecology, and cardiothoracic surgery, where it has been shown to significantly reduce major hemorrhage [5,6,7]. By decreasing the conversion of plasminogen to plasmin, it reduces fibrin degradation but also has an inhibitory effect on several inflammatory processes.

Though the use of TXA in breast surgery is rather novel, and albeit first reports date back to 1994 [17], the majority of studies were published from 2018 onwards [16,43,44], where it has been used both topically [45,46,47] and systemically [17,43,48] with a significant reduction in hematoma and seroma rates but also drainage volume. However, the current literature provides no data for mastectomies with immediate EbR using a standardized systemic TXA protocol.

Systemic administration of TXA using a standardized 48 h protocol significantly reduces drainage volume in patients undergoing NSM, SSM, or SRM with immediate EbR. It was shown that specifically throughout the 48 h period of administration, the difference in drainage volume was markedly reduced in the TXA group (Figure 1), which raises the question of whether patients could benefit from TXA administration beyond 48 h.

While other studies demonstrated a reduction in drainage volume with a systemic 5-day protocol [17,18,49], they are not specific to mastectomy with EbR.

Both duration of drain placement and length of hospital stay were significantly reduced in the TXA group, with a mean decrease of 1.3 and 0.9 days, respectively, potentially greatly impacting patient comfort and health care costs.

Factors associated with an increased drainage volume included hematoma, resection weight of the mammary gland, axillar lymph node dissection ad continuitatem, i.e., when performed from the same surgical approach as the mastectomy. Increased resection weight is associated with a larger wound surface, and axillar lymph node dissection is associated with the transection of lymph vessels discharging into the mastectomy pocket, resulting in increased drainage volume, prolonged drain placement, and eventually longer hospital stay.

Seroma and hematoma rates did not differ between the two groups. With an incidence of hematoma of 6% in the TXA group and 5.5% in the control group, our results are comparable to the literature, where an incidence of 2.3–3.3% is reported [19,20,21]. However, our seroma rates of 6% in the TXA group and 4% in the control group are significantly below the incidence reported in the current literature, ranging from 12% to 44% [17,46,49]. Recent data from 2023 report a seroma incidence of 1.9% in both TXA and control groups undergoing mastectomy with immediate implant-based reconstruction [47]. As the diagnosis “seroma” is not clearly defined, its diagnosis is somewhat subjective and leads to inconsistent descriptions, making comparability difficult.

As mentioned, other studies have investigated different aspects of TXA administration in breast surgery. Three studies investigated the topical application of TXA after mastectomy without immediate reconstruction [46,47,50]. Eldesouky et al. and Ausen et al. have shown decreased drainage volume in patients receiving TXA locally [46,50]; however, only Safran et al. describe immediate reconstructive efforts following the mastectomy, as demonstrated in a randomized control trial on patients undergoing bilateral NSM with immediate implant-based reconstruction, where one breast served as control, and the other was treated with 3 g of TXA diluted in 100 cc of saline. They have shown that topical TXA application would significantly lower drainage volume [47]. However, their study protocol and that of Eldesouky et al. relied on data collected by patients themselves after prompt discharge following surgery [46,47] and, therefore, represents a risk of bias.

Five studies have investigated systemic administration of TXA in patients undergoing breast surgery [17,18,48,49,51]; however, only two were specific to mastectomy [49,51] and, of these, only Gogna et al. measured drainage volume [49].

Oertli et al. investigated the effects of a systemic 5-day regimen of 3 × 1 g TXA daily in patients undergoing both lumpectomies and mastectomies. This resulted in a significant reduction in drainage volume and length of hospital stay [17]. However, the data are not specific to mastectomies, and no subsequent immediate reconstructive procedures are mentioned [17].

Weissler et al. investigated the effect of TXA on complication rates in patients undergoing SSM or NSM, followed by immediate expander-to-implant reconstruction. While administration of 1 g of TXA systemically once prior and once immediately after surgery reduced the risk of hematoma, drainage volume was not assessed [48].

Gogna et al. conducted a prospective randomized study on patients undergoing modified radical mastectomy without reconstruction. They applied a 5-day protocol of 1 g TXA systemically every 8 h and demonstrated a reduction in drainage volume and duration of drain remaining in situ but an increase in post-operative seroma rates [49]. Furthermore, the total time of drains remaining was relatively long, with a mean of 10 days for the TXA group and 13 days for the control group, associated with large amounts of total drainage volume (means of 781 mL and 1023 mL, respectively). Another limitation of this study might be the rather small cohort of 25 patients per group.

The herein presented results not only address this gap but directly translate into clinical practice, where a reduction in drainage volume and, consequently, duration of drains remaining in situ minimizes the risk of further complications like surgical site infections [52,53,54] and allows the patient to return to their normal life sooner. Furthermore, these results show that in patients with minimal risk factors for increased drainage volume (small mastectomy weight and no axillar lymph node dissection ad continuitatem), TXA administration resulted in early drain removal, which is known to further reduce drainage volume, healing time, and experienced pain [55] or might even question the need of drain placement in the first place.

The limitations of this study lie in its retrospective nature. There is a significant difference between case and control groups regarding expander type and expander pocket used. This is due to a change in regimen after June 2019 to not only include TXA after mastectomy but also prefer the prepectoral approach and the use of an MR-compatible expander [56]. This is particularly significant in the context of postmastectomy radiation therapy [57] and radiological follow-up using the hybrid approach where repeated injection of autologous fat graft before exchanging the expander with a definitive implant requires the expander to be in place for a much longer period [58]. The majority of the patients in the control group received a macrotextured expander using a subpectoral placement, whereas the majority of patients in the TXA group received a nanotextured expander mainly placed prepectorally. Although this limits the informative value of the exact estimates, it does not have any negative impact on the finding that TXA reduces drainage volume. Macrotexturization is associated with reduced fluid production in the surgical site due to quicker adhesion to the surrounding tissues, while nanotexturization could lead to less adhesion to the surrounding tissue since they are associated with a longer period of residual fluid staying between the expander and the mastectomy flap resulting in increased or prolonged drainage [59,60]. This may lead to an underestimation of the reduction in drainage volume in the TXA group. The role of the placement is another possible bias; however, current literature, albeit not very extensive, shows no significant difference in drainage volume between pre- and subpectoral placement [61,62]. Additionally, the use of a biological mesh (acellular dermal matrix) was only present in the TXA group, presenting a possible source of bias. Whilst biological meshes are known to influence seroma formation [63], the current study did not show any differences between the two groups. Yet, drainage volume and other associated variables could also be affected. To analyze this, the individual factor of biological mesh was included in the multiple regression analysis, which has shown, however, to be insignificant, which is in line with previous scientific evidence [36].

Nevertheless, the fact that TXA still showed significant improvement in reducing drainage volume despite other counteracting factors is a strong argument in its favor.

Nanotextured expanders were a significant reducing factor throughout all outcome variables, which, however, cannot be ignored and poses the question of the underlying cause, which should be investigated in future studies.

Post-operative thromboembolic events were not included in this study; however, the authors do not recall any thromboembolic events in the first month post-operatively. Although it is known that systemic administration of TXA leads to higher systemic concentrations [64], previous studies have shown that TXA does not increase the risk of thromboembolic events if administered in dosages comparable to this study and respecting the given contraindications [16,44]. Nonetheless, one needs to mention that the administration of TXA in plastic surgery remains an off-label use.

## 5. Conclusions

This study shows that even with factors like low surface texturization of the device, which is associated with increased and prolonged fluid production, TXA still significantly reduces drainage volume. Furthermore, the duration of drain placement and length of hospital stay were reduced in the TXA group, representing the positive impact of TXA on both patient comfort and potential socioeconomic burden. Finally, resection weight of breast tissue and axillar lymph node dissection ad continuitatem with the mastectomy pocket have an important increasing effect on the duration of drain placement and length of hospital stay and could be taken into account when considering drain removal.

This study further supports the systemic use of TXA in reconstructive breast surgery but should also encourage surgeons to reconsider using surgical drains in post-operative protocols for selected surgical indications and patients. Future studies are warranted with health care cost analysis to further promote and bring forward TXA administration in the field of reconstructive breast surgery.

## Figures and Tables

**Table 1:** Baseline patient demographics.

Variable	TXA *N* = 83	Controls *N* = 72	*p*-Value
Age ^a^	50 (46, 58)	48 (42, 62)	0.722 ^d^
BMI ^a^	22.0 (20.8, 26.0)	22.3 (20.3, 25.3)	0.380 ^d^
DM ^b^	1 (1.4)	0 (0)	0.281 ^c^
Hypertension ^b^	10 (12.1)	6 (8.3)	0.448 ^c^
Heart disease ^b^	7 (8.4)	5 (6.9)	0.729 ^c^
Kidney disease ^b^	4 (4.8)	0 (0)	0.059 ^c^
Active smoking ^b^	27 (32.5)	17 (23.6)	0.219 ^c^
Chronic NSAID ^b^	2 (2.8)	1 (1.2)	0.478 ^c^
Antiaggregation ^b^	1 (1.2)	4 (5.6)	0.126 ^c^
Anticoagulation ^b^	2 (2.4)	1 (1.4)	0.645 ^c^
Previous thromboembolic event ^b^	0 (0)	4 (5.6)	0.030 ^c^
Previous radiotherapy ^b^	7 (8.4)	0 (0)	0.012 ^c^
Neoadjuvant chemotherapy ^b^	21 (25.3)	11 (15.3)	0.124 ^c^
Thrombocytes ^a^	253 (220, 312)	269 (219, 331)	0.236 ^d^
Quick ^a^	103 (96, 113)	98.5 (90, 106)	0.012 ^d^
INR ^a^	1 (0.9, 1)	1 (1, 1)	0.002 ^d^

^a^ Median (25th percentile, 75th percentile), ^b^ N (%), ^c^ Chi^2^-test, ^d^ *t*-test, BMI = body mass index, DM = diabetes mellitus, NSAID = non-steroidal anti-inflammatory drugs, INR = international normalized ratio.

**Table 2:** Surgical parameters.

Variable	TXA *N* = 83	Controls *N* = 72	*p*-Value
Breast			
Right ^b^	41 (49.4)	30 (41.7)	0.335 ^c^
Left ^b^	42 (50.6)	42 (58.3)	0.335 ^c^
Resection weight (g) ^a^	332 (228, 550)	316.5 (218.5, 485.5)	0.588 ^d^
Type of mastectomy			<0.001 ^c^
Skin-sparing ^b^	14 (16.9)	23 (31.9)	
Nipple-sparing ^b^	36 (43.4)	45 (62.5)	
Skin-reducing ^b^	33 (39.8)	4 (5.6)	
Expander placement			<0.001 ^c^
Prepectoral ^b^	71 (85.5)	29 (40.3)	
Subpectoral ^b^	12 (14.5)	43 (59.7)	
Expander			<0.001 ^c^
Allergan (Natrelle 133) ^b^	0 (0)	65 (90.3)	
Mentor (CPX4) ^b^	18 (21.7)	5 (6.9)	
Motiva (Flora) ^b^	65 (78.3)	2 (2.8)	
Sentinel lymph node biopsy ^b^	49 (59.0)	49 (68.1)	0.245 ^c^
Axillar lymph node dissection ad continuitatem^b^	6 (7.2)	6 (8.3)	0.797 ^c^
Axillar access ^b^	39 (47.0)	41 (56.9)	0.216 ^c^
Use of synthetic mesh ^b^	10 (12.1)	14 (19.4)	0.204 ^c^
Use of biological mesh (acellular dermal matrix) ^b^	11 (13.3)	0 (0)	0.001 ^c^

^a^ Median (25th percentile, 75th percentile), ^b^ N (%), ^c^ Chi^2^-test, ^d^ *t*-test.

**Table 3:** Surgical complications and secondary outcome measures.

Variable	TXA *N* = 83	Controls *N* = 72	*p*-Value
Hematoma ^b^	5 (6.0)	4 (5.6)	0.901 ^c^
Seroma ^b^	5 (6.0)	3 (4.2)	0.602 ^c^
Duration of drain placement (days) ^a^	3.3 (1.8)	4.6 (1.6)	<0.001 ^d^
Length of hospital stay (days) ^a^	5.3 (1.8)	6.2 (1.5)	0.001 ^d^

^a^ Mean (SD), ^b^ N (%), ^c^ Chi^2^-test, ^d^ *t*-test.

**Table 4:** Full Model—drainage volume.

	Univariate Analysis			Multivariate Analysis		
Variable	Estimate (SE)	*p*-Value	95% CI	Estimate (SE)	*p*-Value	95% CI
Age (per year)	0.1 (0.2)	0.520	−0.2–0.5	0.1 (0.1)	0.401	−0.2–0.4
BMI (per kg/m^2^)	0.9 (0.5)	0.098	−0.2–2.0	0.3 (0.6)	0.607	−0.9–1.5
Current smoking	−7.1 (4.6)	0.118	−16.1–1.8	−4.4 (3.7)	0.234	−11.5–2.8
Type of mastectomy (skin-sparing as reference)						
Nipple-sparing	−9.0 (5.0)	0.069	−18.8–0.7	−3.6 (4.1)	0.387	−11.7–4.5
Skin-reducing	−17.0 (5.8)	0.004	−28.4–(−5.5)	−10.8 (5.1)	0.034	−20.8–(−0.8)
Biologic mesh (acellular dermal matrix)	−18.6 (7.9)	0.018	−34.1–(−3.2)	−2.7 (6.6)	0.690	−15.7–10.4
Type of expander (Mentor (CPX4) as reference)						
Allergan (Natrelle 133)	1.9 (5.4)	0.729	−8.8–12.5	−18.4 (8.1)	0.023	−34.3–(−2.5)
Motiva (Flora)	−25.0 (5.4)	<0.001	−35.6–(−14.4)	−23.5 (5.0)	<0.001	−33.3–(−13.8)
Axillar lymph node dissection ad continuitatem	20.3 (7.7)	0.009	5.2–35.4	18.3 (5.8)	0.002	6.9–29.7
Resection weight (per gram)	0.03 (0.01)	0.002	0.01–0.05	0.04 (0.01)	0.001	0.01–0.06
Hematoma	20.2 (8.7)	0.020	3.2–37.3	23.7 (6.7)	<0.001	10.6–36.8
Tranexamic acid	−23.3 (3.7)	<0.001	−30.6–(−16.1)	−19.0 (8.1)	0.020	−35.0–(−3.0)

BMI = body mass index.

**Table 5:** Full Model—duration of drain placement.

	Univariate Analysis			Multivariate Analysis		
Variable	Estimate (SE)	*p*-Value	95% CI	Estimate (SE)	*p*-Value	95% CI
Age (per year)	−0.004 (0.01)	0.759	−0.03–0.02	−0.01 (0.01)	0.468	−0.03–0.02
BMI (per kg/m^2^)	0.1 (0.04)	0.033	0.007–0.2	0.0 (0.1)	0.995	−0.1–0.1
Current smoking	−0.7 (0.3)	0.050	−1.3–(−0.001)	−0.5 (0.3)	0.148	−1.1–0.2
Type of mastectomy (skin-sparing as reference)						
Nipple-sparing	−0.7 (0.4)	0.062	−1.5–0.04	−0.3 (0.4)	0.360	−1.0–0.4
Skin-reducing	−0.9 (0.4)	0.042	−1.8–(−0.04)	−0.5 (0.4)	0.236	−1.4–0.3
Biologic mesh (acellular dermal matrix)	−1.3 (0.6)	0.021	−2.5–(−0.2)	−0.5 (0.6)	0.399	−1.6–0.6
Type of expander (Mentor (CPX4) as reference)						
Allergan (Natrelle 133)	0.1 (0.5)	0.753	−0.7–1.0	−0.7 (0.7)	0.313	−2.0–0.7
Motiva (Flora)	−1.3 (0.5)	0.004	−2.2–(−0.4)	−1.3 (0.5)	0.004	−2.2–(−0.4)
Axillar lymph node dissection ad continuitatem	1.2 (0.6)	0.036	0.08–2.3	1.0 (0.5)	0.055	−0.02–2.0
Resection weight (per gram)	0.003 (0.001)	<0.001	0.002–0.004	0.003 (0.001)	0.001	0.001–0.005
Hematoma	1.1 (0.7)	0.105	−0.2–2.4	1.2 (0.6)	0.049	0.005–2.3
Tranexamic acid	−1.3 (0.3)	<0.001	−1.9–(−0.7)	−0.7 (0.7)	0.341	−2.0–0.7

BMI = body mass index.

**Table 6:** Full Model—length of hospital stay.

	Univariate Analysis			Multivariate Analysis		
Variable	Estimate (SE)	*p*-Value	95% CI	Estimate (SE)	*p*-Value	95% CI
Age (per year)	0.005 (0.01)	0.720	−0.02–0.03	−0.003 (0.01)	0.819	−0.03–0.02
BMI (per kg/m^2^)	0.1 (0.03)	<0.001	0.1–0.2	0.095 (0.047)	0.043	0.003–0.2
Current smoking	−0.5 (0.3)	0.110	−1.1–0.1	−0.286 (0.290)	0.326	−0.9–0.3
Type of mastectomy (skin-sparing as reference)						
Nipple-sparing	−0.7 (0.3)	0.045	−1.3–(−0.02)	−0.267 (0.330)	0.419	−0.9–0.4
Skin-reducing	−0.6 (0.4)	0.134	−1.4–0.2	−0.513 (0.405)	0.207	−1.3–0.3
Biologic mesh (acellular dermal matrix)	−0.8 (0.5)	0.117	−1.9–0.2	0.097 (0.533)	0.855	−1.0–1.2
Type of expander (Mentor (CPX4) as reference)						
Allergan (Natrelle 133)	−0.005 (0.4)	0.991	−0.8–0.8	−0.782 (0.649)	0.230	−2.1–0.5
Motiva (Flora)	−1.1 (0.4)	0.005	−1.9–(−0.3)	−1.236 (0.391)	0.002	−2.0–(−0.5)
Axillar lymph node dissection ad continuitatem	0.4 (0.5)	0.409	−0.6–1.4	0.248 (0.461)	0.591	−0.7–1.2
Resection weight (per gram)	0.003 (0.001)	<0.001	0.002–0.004	0.002 (0.001)	0.025	0.0–0.004
Hematoma	0.6 (0.6)	0.338	−0.6–1.7	0.965 (0.535)	0.073	−0.1–2.0
Tranexamic acid	−0.9 (0.3)	0.001	−1.4–(−0.4)	−0.611 (0.650)	0.349	−1.9–0.7

BMI = body mass index.

## Data Availability

The data presented in this study are available on request from the corresponding author due to privacy reasons.
